# Targeted gut microbiota manipulation attenuates seizures in a model of infantile spasms syndrome

**DOI:** 10.1172/jci.insight.158521

**Published:** 2022-06-22

**Authors:** Chunlong Mu, Naghmeh Nikpoor, Thomas A. Tompkins, Anamika Choudhary, Melinda Wang, Wendie N. Marks, Jong M. Rho, Morris H. Scantlebury, Jane Shearer

**Affiliations:** 1 Department of Biochemistry and Molecular Biology, Cumming School of Medicine, University of Calgary, Calgary, Alberta, Canada.; 2 Lallemand Bio Ingredients, Lallemand Inc., Montreal, Quebec, Canada.; 3 Department of Paediatrics,; 4 Department of Clinical Neurosciences, Cumming School of Medicine, and; 5 Alberta Children’s Hospital Research Institute, Hotchkiss Brain Institute, University of Calgary, Calgary, Alberta, Canada.; 6 Departments of Neurosciences and Pediatrics, University of California San Diego, Rady Children’s Hospital, San Diego, California, USA.

**Keywords:** Metabolism, Epilepsy, Neurological disorders

## Abstract

Infantile spasms syndrome (IS) is a devastating early-onset epileptic encephalopathy associated with poor neurodevelopmental outcomes. When first-line treatment options, including adrenocorticotropic hormone and vigabatrin, are ineffective, the ketogenic diet (KD) is often employed to control seizures. Since the therapeutic impact of the KD is influenced by the gut microbiota, we examined whether targeted microbiota manipulation, mimicking changes induced by the KD, would be valuable in mitigating seizures. Employing a rodent model of symptomatic IS, we show that both the KD and antibiotic administration reduce spasm frequency and are associated with improved developmental outcomes. Spasm reductions were accompanied by specific gut microbial alterations, including increases in *Streptococcus thermophilus* and *Lactococcus lactis*. Mimicking the fecal microbial alterations in a targeted probiotic, we administered these species in a 5:1 ratio. Targeted probiotic administration reduced seizures and improved locomotor activities in control diet–fed animals, similar to KD-fed animals, while a negative control (*Ligilactobacillus salivarius*) had no impact. Probiotic administration also increased antioxidant status and decreased proinflammatory cytokines. Results suggest that a targeted probiotic reduces seizure frequency, improves locomotor activity in a rodent model of IS, and provides insights into microbiota manipulation as a potential therapeutic avenue for pediatric epileptic encephalopathies.

## Graphical abstract

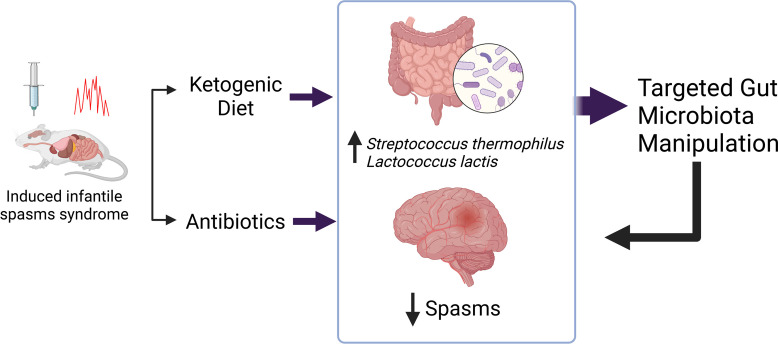

## Introduction

Infantile spasms syndrome (IS), also known as West Syndrome, is a developmental epileptic encephalopathy occurring in the first year of life. Characterized by distinctive spasms, interictal hypsarrhythmia (EEG) and cognitive arrest or regression, spasms often occur upon wakening and in clusters, resulting in hundreds of seizures per day in some children. At this vulnerable age, the condition is devastating to families and has important clinical implications because of frequent seizure recurrence, risk for developing future epilepsy, intellectual disability, and comorbid autism leading to lifelong debilitation (1).

First-line anticonvulsant treatments include pharmacological intervention, usually with adrenocorticotropic hormone (ACTH) or vigabatrin. The efficacy of ACTH and vigabatrin is 55% and 36%, respectively, based on a 3-month assessment in infants (2). In drug-resistant cases, infants are often treated with the ketogenic diet (KD), a high-fat, low-carbohydrate regimen administered as a formula. Retrospective studies suggest that the KD is effective in 30%–60% of children, with pediatric epilepsy defined as a reduction in seizures of at least 50% following 6 months of treatment (3). Likewise, a prospective study showed that the KD is equally as effective as ACTH and has improved tolerability over a 6-month period in IS (4). While the KD can control seizures, it can also have implications for somatic growth, development, gut microbiota, and the potential development of lipid-related disorders (5–7). Given this, there is an urgent and unmet need to develop alternative therapies for IS.

Compelling evidence shows a protective role of the gut microbiota in epilepsy, potentially through regulation of amino acid γ-glutamylation and hippocampal γ-aminobutyric acid production. Specifically, the KD attenuated seizures in both the 6 Hz seizure model (a model for drug-resistant epilepsy) and spontaneously epileptic *Kcna1*-null mice (a validated model of temporal lobe epilepsy) through mechanisms involving in the gut microbiota (8). In humans, a recent clinical trial in adult epilepsy patients showed efficacy of a probiotic mixture against drug-resistant epilepsy, with 29% of patients displaying a greater than 50% reduction in the number of seizures (9). Collectively, these studies highlight the bidirectional interactions between the gut microbiota and the brain in epilepsy, which may provide an alternative microbiota-targeted strategy for treatment (10).

We hypothesized that the gut microbiota may also be involved in regulating seizures in IS and also responsible for the protective impacts of the KD. Employing a well-established neonatal rat model that successfully recapitulates the neurodevelopmental deficits and neuropathology observed in children with symptomatic IS (11, 12), we developed and tested a targeted probiotic specific to IS based on the KD and the young developmental age of the animals.

## Results

### KD reduces seizures, altering the gut microbiota and circulating metabolites.

**Figure 1:**
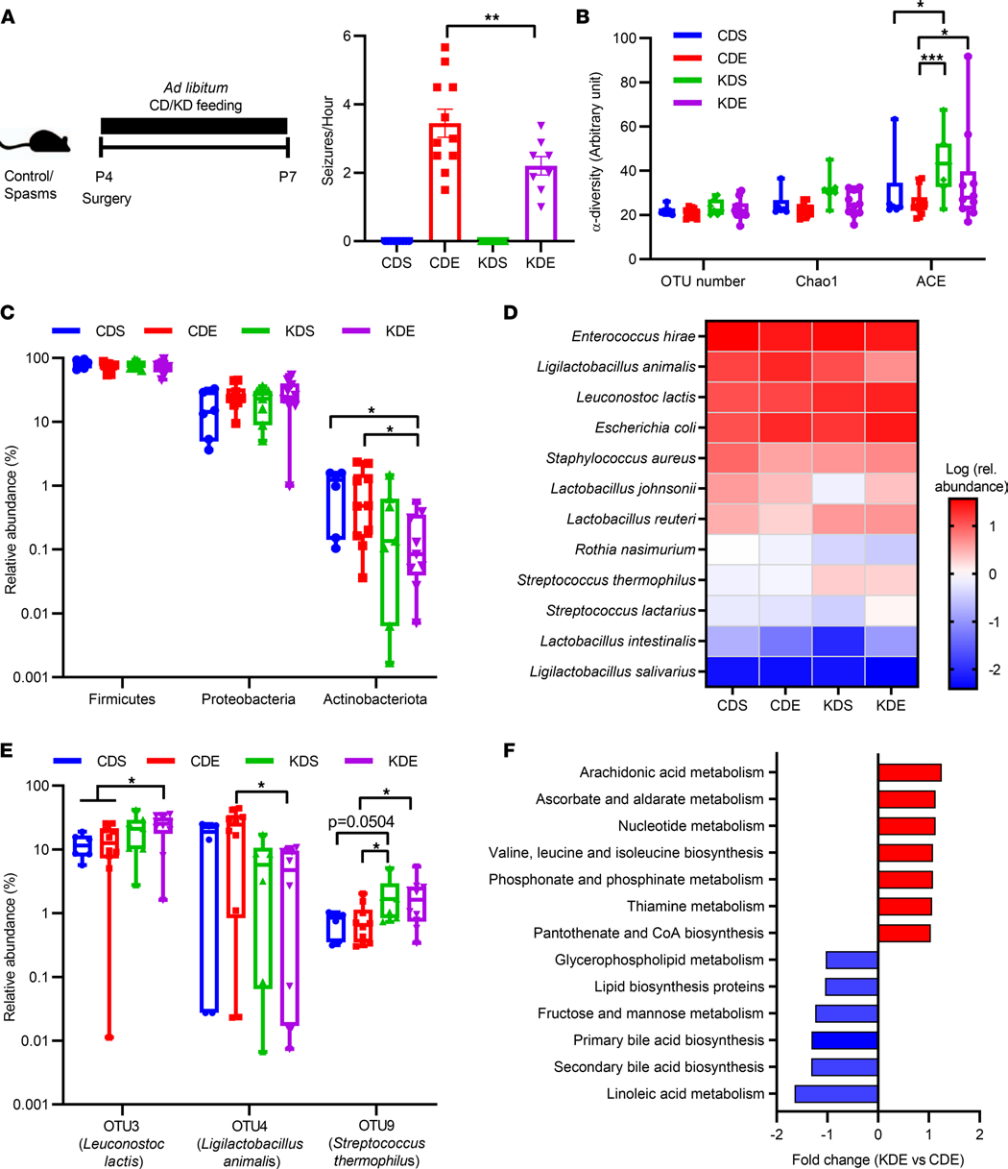
Effects of the ketogenic diet formula on number of seizures and gut microbiota composition. (**A**) Animals were fed with a control or ketogenic formula after sham (CDS, KDS) or epilepsy induction (CDE, KDE) following the timeline shown (left panel); resulting seizure frequency (right panel). (*n* = 9, 11, 9, and 8 for CDS, CDE, KDS, and KDE, respectively). (**B**) α-Diversity. (**C**) Microbial composition at the phylum level. (**D**) Dominant phylotypes in the fecal microbiota. (**E**) Phylotypes affected by the ketogenic formula. (**F**) Comparison of PICRUSt-based metagenomics prediction between CDE and KDE. *n* = 6, 10, 7, and 10 for CDS, CDE, KDS, and KDE, respectively, for **B**–**F**. The data were analyzed using 1-way ANOVA with Tukey’s post hoc (**A** and **B**) or Kruskal-Wallis 1-way ANOVA with Dunn’s post- hoc test (**C** and **E**). (**A**) Values are mean ± SEM. (**B**, **C**, and **E**) The data are presented as box and whisker plots (minimum to maximum values). *P < 0.05, **P < 0.01, ***P < 0.001.

To investigate the effects of KD on mitigating spasms, we employed a neonatal rodent model of IS (11, 12). Neonatal rats were intracerebrally injected with doxorubicin and LPS at P4, and i.p. injected with chlorophenylalanine at P5 to induce spasms. Four experimental treatment groups were examined: control diet saline (CDS) injected; control diet epilepsy (CDE) induction; KD saline injected (KDS); and KD epilepsy induction (KDE). Compared with the CDE, KDE significantly reduced seizure frequency (*P* = 0.003, Figure 1A).

To explore gut microbiota alterations with the diets, we next analyzed fecal microbiota structures. The Abundance-based Coverage Estimator (ACE) index represents the community richness of the gut microbiota, which is a parameter of α-diversity. The KD increased the ACE index in KDE compared with CDE groups (*P* = 0.023, Figure 1B). The difference was driven by several samples with elevated values, indicating high variation within the KDE groups. Diet had significant impact on the relative abundance of Actinobacteria (*P*_diet_ = 0.003, Figure 1C). The KD reduced relative abundances of Actinobacteria (*P* = 0.029 for KDE versus CDE, Figure 1C). Relative abundances of dominant species are presented in Figure 1D, including *Enterococcus hirae*, *Ligilactobacillus animalis* (previously described as *Lactobacillus animalis*), and *Streptococcus* *thermophilus*. There were significant effects of diet on relative abundances of *Leuconostoc lactis* (*P*_diet_ = 0.002)*, S*. *thermophilus* (*P*_diet_ = 0.009), and *L*. *animalis* (*P*_diet_ = 0.009, Figure 1E). In IS animals, the KD increased relative abundances of *L. lactis* (*P* = 0.044) and *S*. *thermophilus* (*P* = 0.022) while decreasing those of *L*. *animalis* (*P* = 0.017) compared with animals fed the control diet (CDE) (Figure 1E). To gain insight into alterations in microbial functions affected by the KD, microbiota functional potential was explored in the IS groups. Results showed an upregulation of arachidonic acid metabolism and vitamin and cofactor metabolism including ascorbate, thiamine, and pantothenate with KD (*P* < 0.05, Figure 1F). Given that ascorbate and thiamine are well-known antioxidants in vivo, they may affect both seizure susceptibility and systemic metabolism.

**Figure 2:**
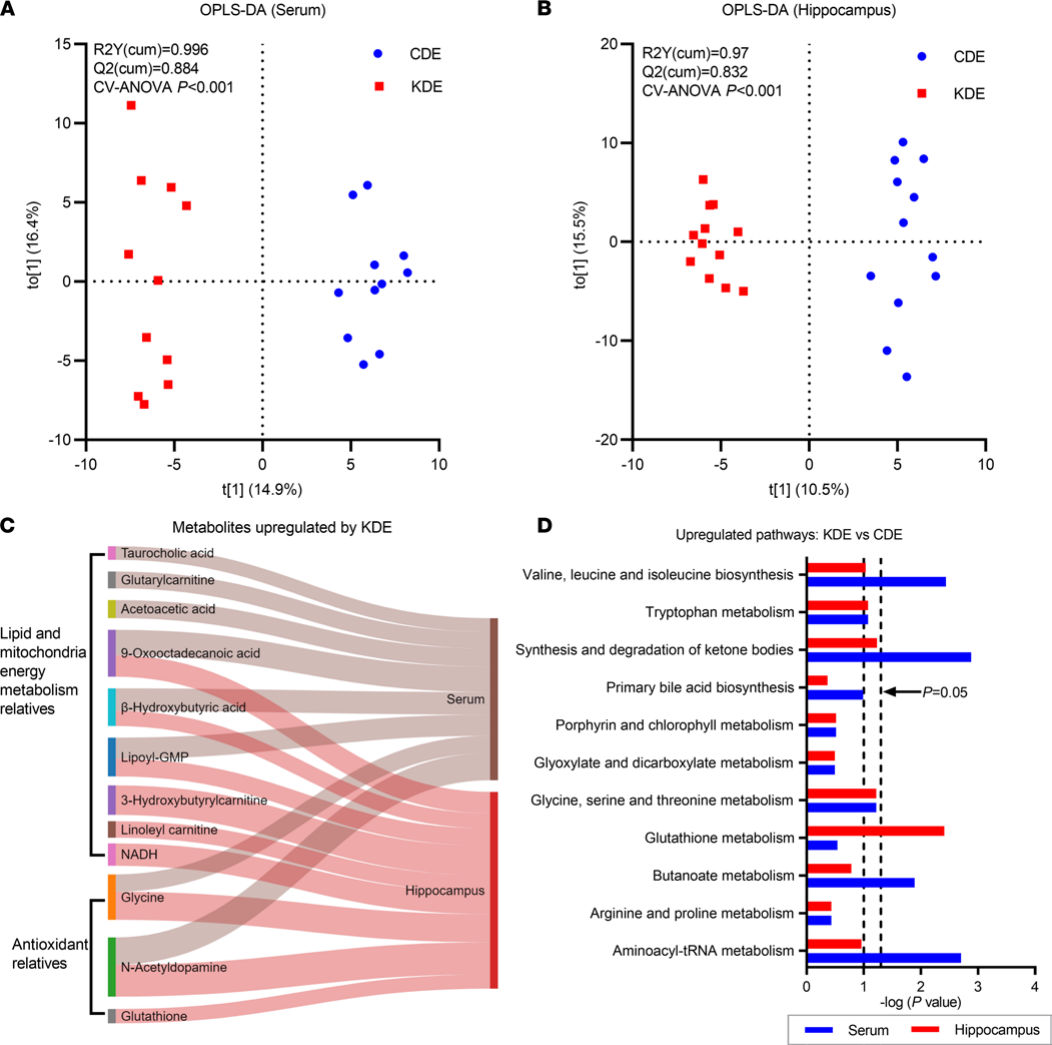
Metabotype shifts following ketogenic diet formula administration. (**A** and **B**) Orthogonal Projections to Latent Structures Discriminant Analysis (OPLS-DA) analysis of metabolite composition in serum (**A**) and hippocampus (**B**). (**C**) Sankey diagram shows the representative metabolites altered by the ketogenic formula in serum and hippocampus. Metabolites with VIP >1 are shown. (**D**) Pathway analysis of the metabolites upregulated by KDE versus CDE. *n* = 11 per group for serum, and *n* = 12 per group for hippocampus.

To investigate if KD-induced microbiota alterations were paralleled in peripheral and central metabolic shifts, samples from CDE and KDE were used for metabolomics profiling. The KDE led to a distinct metabolic status both in serum (cross-validated ANOVA [CV-ANOVA], *P* < 0.001) and hippocampus (CV-ANOVA, *P* < 0.001) compared with CDE, as shown in Orthogonal Projections to Latent Structures Discriminant Analysis (OPLS-DA) plots (Figure 2, A and B). Among the discriminant metabolites, we identified an increase of lipid and mitochondria energy metabolism relatives (β-hydroxybutyric acid, lipoyl-GMP), and antioxidant relatives (glycine, *N*-acetyldopamine) in both serum and hippocampus (variable importance of projection [VIP] > 1, *P* < 0.05, Figure 2C). Additionally, the KD increased taurocholic acid, glutarylcarnitine, acetoacetic acid in the serum, and 3-hydroxybutyrylcarnitine, linoleyl carnitine, glutathione in the hippocampus (VIP > 1, *P* < 0.05, Figure 2C). Pathway analysis further identified the numerical upregulation of several pathways involved in branched-chain amino acid, ketone, and glutathione metabolism (*P* < 0.05, Figure 2D). Similar upregulation of serum β-hydroxybutyric acid, glycine, and taurocholic acid have also been observed in an adult mouse model of epilepsy (8). Collectively, these results support shifts in the metabolic phenotype of animals following KD treatment.

### Antibiotics further mitigate seizures and are additive with the KD.

**Figure 3:**
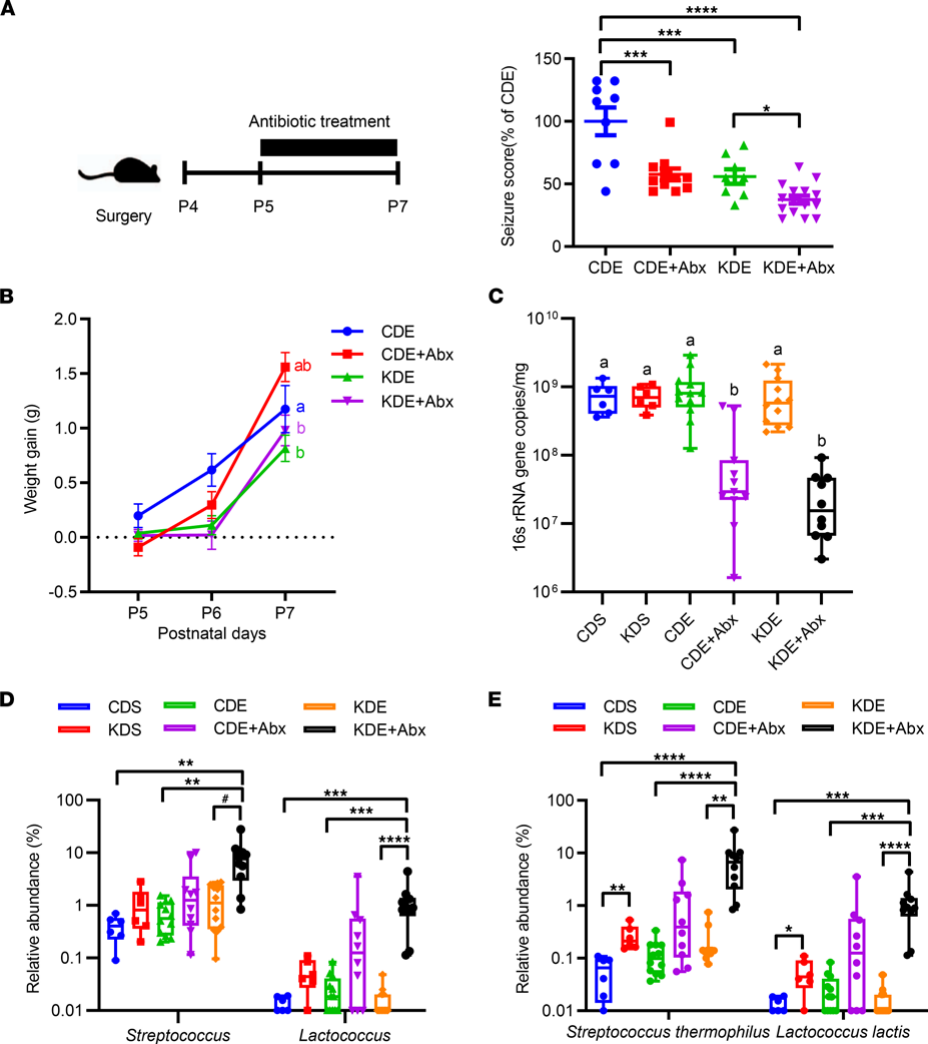
Effects of antibiotics on the gut microbiota composition. (**A**) Animals were given diets without (CDE, KDE) or with antibiotics (CDE + Abx; KDE + Abx) after epilepsy induction from P5 to P7 following the timeline shown (left); effect of antibiotic treatment on seizure frequency (right). (*n* = 9, 11, 8, and 15 for CDE, CDE + Abx, KDE, and KDE + Abx, respectively). The data were analyzed using 1-way ANOVA with Tukey’s post hoc. (**B**) Body weight gain (*n* = 14, 17, 20, and 17 for CDE, CDE + Abx, KDE, and KDE + Abx, respectively). (**C**) Quantitative PCR analysis of fecal 16S rRNA gene copies (*n* = 6, 6, 12, 11, 12, and 10 for CDS, KDS, CDE, CDE + Abx, KDE, and KDE + Abx, respectively). (**D**) Relative abundances of *Streptococcus* and *Lactococcus* (*n* = 6, 6, 12, 10, 12, and 10 for CDS, KDS, CDE, CDE + Abx, KDE, and KDE + Abx, respectively). (**E**) Relative abundances of *Streptococcus* *thermophilus* and *Lactococcus lactis* (*n* = 6, 6, 12, 11, 12, and 10 for CDS, KDS, CDE, CDE + Abx, KDE, and KDE + Abx, respectively). (**A** and **B**) values are mean ± SEM. (**C**–**E**) The data are presented as box and whisker plots (minimum to maximum values). (**A**, **D**, and **E**) *P < 0.05, **P < 0.01, ***P < 0.001, ****P < 0.0001, ^#^0.1 < *P* < 0.05. (**B** and **C**) Labeled means without a common superscript letter differ, *P* < 0.05. CDE, CDE + Abx are control diet, epilepsy induction without and with antibiotics; KDE, KDE + Abx are ketogenic diet, epilepsy induction without and with antibiotics.

Since seizure frequency is influenced by antibiotic use (13), we used this experimental approach to investigate the association between the gut microbiota and the impacts of the KD in IS. To deplete the gut microbiota, epileptic animals were exposed to a cocktail of broad-spectrum antibiotics (Abx) in their formula. Results show that antibiotics further increased KD efficacy in decreasing seizure scores (*P* < 0.001 for KDE + Abx versus CDE, *P* = 0.039 for KDE + Abx versus KDE) (Figure 3A). Both diet and antibiotics affected the seizure scores (*P*_diet_ < 0.001, *P*_Abx_< 0.001) (Figure 3A). Antibiotic treatment resulted in the CDE group having greater weight gain than KDE (*P* =0.055) and KDE + Abx (*P* = 0.099, Figure 3B). In CDE, antibiotics increased the relative liver weight compared with their untreated counterparts (*P* = 0.002, Supplemental Figure 1A; supplemental material available online with this article; https://doi.org/10.1172/jci.insight.158521DS1). Relative to CDE + Abx, antibiotics in KDE animals showed higher brain weight (*P* = 0.05) but lower liver weight (*P* = 0.023, Supplemental Figure 1A). The antibiotic treatment regimen reduced the fecal bacteria load in both CD-fed (*P* = 0.016, CDE + Abx versus CDE) and KD-fed animals (*P* < 0.001, KDE + Abx versus KDE; Figure 3C). KDE + Abx animals had the highest α-diversity, as indicated by the increased number of operational taxonomic units (OTUs; *P* < 0.001 for KDE + Abx versus CDE) and the Chao1 index (*P* < 0.001 for KDE + Abx versus CDE) (Supplemental Figure 1B). No differences were observed at the phylum level (Supplemental Figure 1C). At the genus level, antibiotics exerted similar effects in CDE and KDE, including increases in the relative abundances of *Streptococcus* (*P* = 0.002 for KDE + Abx versus CDE) and *Lactococcus* (*P* < 0.001 for KDE + Abx versus CDE) and a decrease in *Lactobacillus* (*P* = 0.077 for KDE + Abx versus CDE) and *Enterococcus* (*P* = 0.015 for KDE + Abx versus CDE) (Figure 3D and Supplemental Figure 1D). There was a statistically significant 3-way interaction between diet, epilepsy, and antibiotics on *Streptococcus* (*P*_interaction_ < 0.001) and *Lactococcus* (*P*_interaction_ = 0.001) (Figure 3D). Overall, the antibiotic treatment reduced seizure occurrence and changed the fecal microbiota.

### Targeted gut microbiota manipulation for IS.

**Figure 4:**
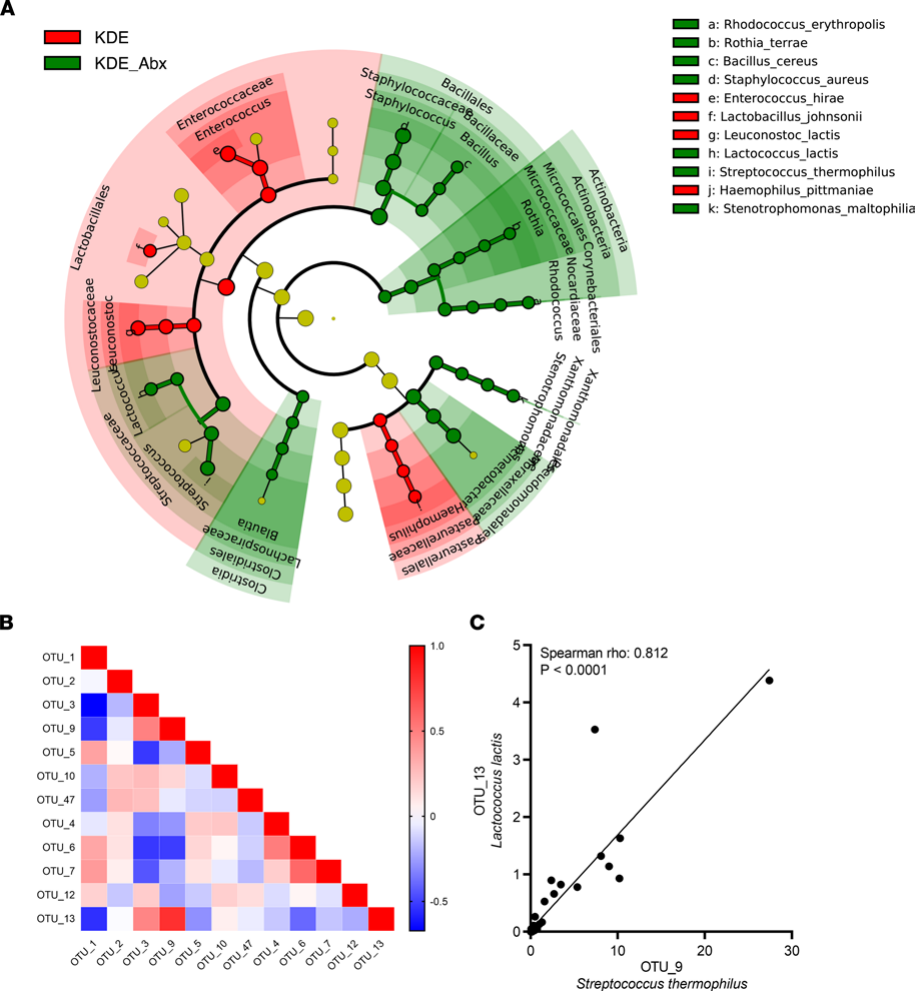
Effects of antibiotics on the gut microbial composition at the species level and correlations between species. (**A**) A linear discriminant analysis effect size (LEfSe) algorithm of microbiota comparing KDE and KDE + Abx. Taxa with logarithmic discriminant analysis score >2 are presented. (**B**) Correlation matrix of dominant OTUs (Spearman’s rank correlation). (**C**) A significant positive correlation between *Streptococcus* *thermophilus* and *Lactococcus lactis*. KDE, KDE + Abx are ketogenic diet, epilepsy induction without and with antibiotics; OTUs, operational taxonomic units.

It is likely that both the KD and antibiotic interventions eliminated bacterial species or metabolic by-products that may affect seizures — or that they enhanced the growth of others that reduced seizures. To this end, we sought common microbes that increased in both the KD and antibiotic treatments, both of which significantly reduced seizure frequency. We chose candidates that changed similarly with both treatments and were known psychobiotics, species known to influence the gut-brain axis (14, 15). Among the common species affected by the KD and antibiotics, we noted the remarkable increase of *S*. *thermophilus* from 0.2% to 7.80% (39-fold, *P* < 0.001) and *L*. *lactis* from < 0.01% to 1.22% (>100 fold, *P* < 0.001) (Figure 3E). There was a statistically significant 3-way interaction between diet, epilepsy, and antibiotics on *S*. *thermophilus* (*P*_interaction_ < 0.001) and *L*. *lactis* (*P*_interaction_ = 0.001) (Figure 3E). Quantification of bacterial 16S rRNA gene copies showed that, although the counts of *S*. *thermophilus* and *L*. *lactis* were reduced after antibiotics, the abundances inferred from the gene copies (copies per taxa divided by total 16S rRNA gene copies) were increased, a finding consistent with our sequencing results (Supplemental Figure 2). The differences were further confirmed when comparing KDE and KDE + Abx using a linear discriminant analysis effect size (LEfSe) algorithm (linear discriminant analysis [LDA] score > 2, Figure 4A). We then constructed the correlation matrix between species (Figure 4B). A significant positive correlation was identified between OTU9 and OTU13 (Spearman’s ρ = 0.812, *P* < 0.001), corresponding to *S*. *thermophilus* and *L*. *lactis* (Figure 4C), respectively, indicating significant cooccurrence. Both *S*. *thermophilus* and *L*. *lactis* have been used as parts of probiotic mixtures but have never been used in combination (15). Based on their abundance in the feces following KD and antibiotic treatments, we administered *S*. *thermophilus* and *L*. *lactis* at a 5:1 ratio in the subsequent probiotic study. *L*. *salivarius* HA-118 (Lsa) was used as a negative control bacterium as previously described (16). Of note, the relative abundance of *L*. *salivarius* was not affected by the KD in the present study.

### Impact of a targeted or negative control probiotic in a model of IS.

**Figure 5:**
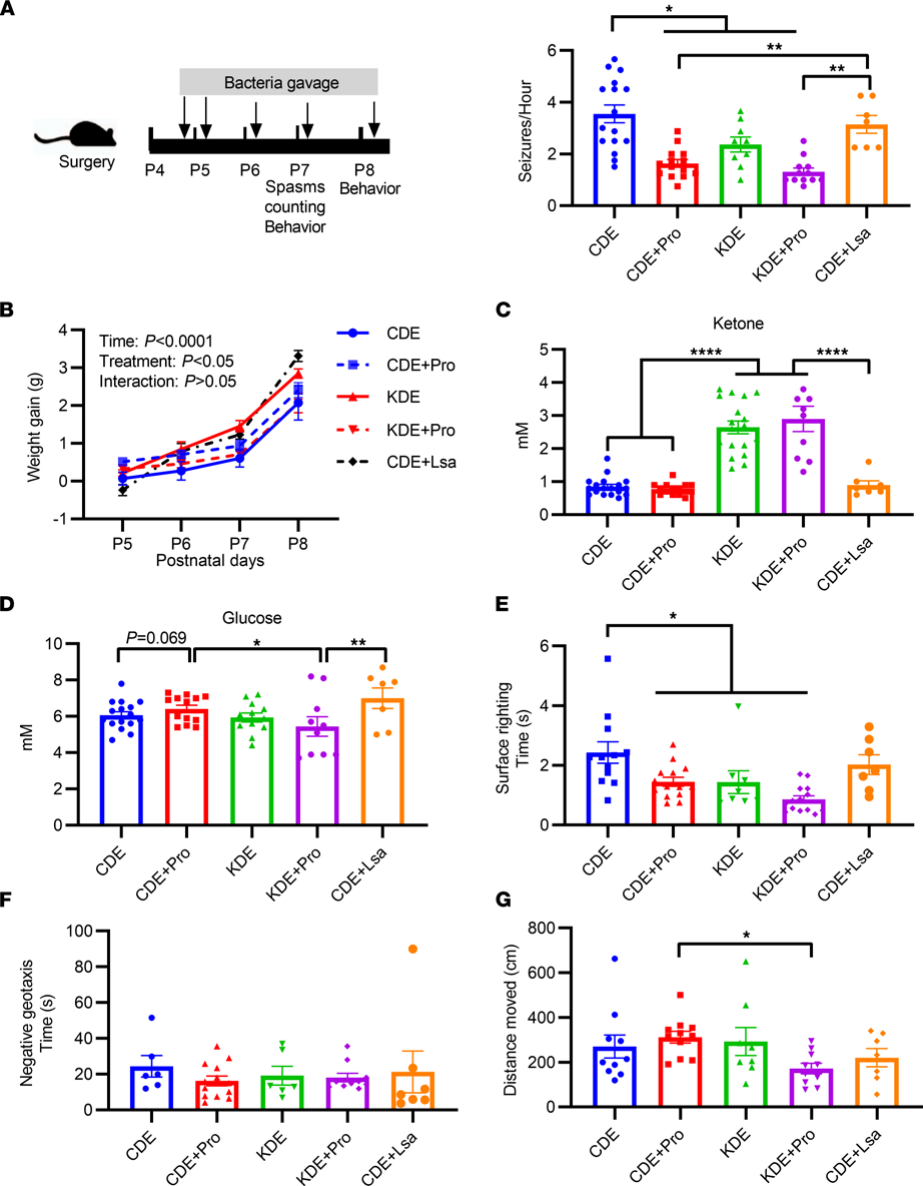
Effects of targeted and negative control probiotic administration on seizures and behaviors. (**A**) Targeted probiotics (Pro) were administered after epilepsy induction following the timeline shown (left panel); effects of targeted probiotic treatment on seizure frequency (right). (*n* = 16, 14, 9, 12, and 7 for CDE, CDE + Pro, KDE, KDE + Pro, and CDE + Lsa, respectively). (**B**) Body weight gain (*n* = 10, 13, 9, 12, and 7 for CDE, CDE + Pro, KDE, KDE + Pro, and CDE + Lsa, respectively). Two-way ANOVA with repeated-measure analysis was applied to compare the effects of age or treatment on the body weight gain. (**C**) Blood ketone concentration (*n* = 18, 15, 18, 10, and 7 for CDE, CDE + Pro, KDE, KDE + Pro, and CDE + Lsa, respectively). (**D**) Blood glucose concentration (*n* = 16, 13, 12, 10, and 7 for CDE, CDE + Pro, KDE, KDE + Pro, and CDE + Lsa, respectively). (**E**) Surface righting time (*n* = 12, 14, 8, 14, and 7 for CDE, CDE + Pro, KDE, KDE + Pro, and CDE + Lsa, respectively). (**F**) Negative geotaxis (*n* = 6, 13, 6, 10, and 7 for CDE, CDE + Pro, KDE, KDE + Pro, and CDE + Lsa, respectively). (**G**) Open field activities (*n* = 10, 11, 8, 10, and 7 for CDE, CDE + Pro, KDE, KDE + Pro, and CDE + Lsa, respectively). The data were analyzed using 1-way ANOVA with Tukey’s post hoc (**A** and **C**–**G**). Values are mean ± SEM. *P < 0.05, **P < 0.01, ****P < 0.0001. CDE, control diet, epilepsy induction; CDE + Pro, CDE with probiotics; KDE, ketogenic diet, epilepsy induction; KDE + Pro, KDE + probiotics; Lsa, *L*. *salivarius* negative control.

Next, the impact of the targeted probiotic in the IS model was investigated. Compared with CDE animals, the inclusion of the probiotic significantly reduced seizure frequency to levels observed in the KDE group (*P* < 0.001 versus CDE, Figure 5A). In contrast, administration of the negative control, *L*. *salivarius*, had no effect on spasm occurrence (*P* = 0.874 for CDE + Lsa versus CDE). Of interest, body mass showed both time-dependent (*P* < 0.001) and treatment-dependent effects (*P* = 0.042, Figure 5B) with the greatest gain observed in the CDE + Lsa group. As anticipated, KDE and KDE + Probiotics (Pro) animals had higher blood ketone concentrations than the other groups (*P* < 0.001, Figure 5C). No differences were observed for CDE + Pro and CDE animals (*P* = 0.997), suggesting that the effects of the probiotic were likely ketone independent (Figure 5C). CDE + Pro rats tended to exhibit increased blood glucose concentrations compared with CDE animals (*P* = 0.069, Figure 5D). Similar elevations were also observed in the CDE + Lsa group. Behavior analysis showed CDE + Pro (*P* = 0.005), KDE (*P* = 0.014), and KDE + Pro rats (*P* < 0.001) had shorter surface righting times compared with CDE animals (Figure 5E), while no differences were observed for the negative geotaxis test (ANOVA, *P* = 0.866, Figure 5F). In the open field activity test, KDE + Pro animals had a decrease in the distance travelled compared with the CDE + Pro group (*P* = 0.015, Figure 5G). There was a statistically significant 3-way interaction between diet, probiotics and negative strain on spasms (*P*_interaction_ < 0.001, Figure 5A), ketone concentration (*P*_interaction_ < 0.001, Figure 5C), and surface righting time (*P*_interaction_ = 0.013, Figure 5E).

### Probiotics shift the gut microbiota and alter host metabolism.

**Figure 6:**
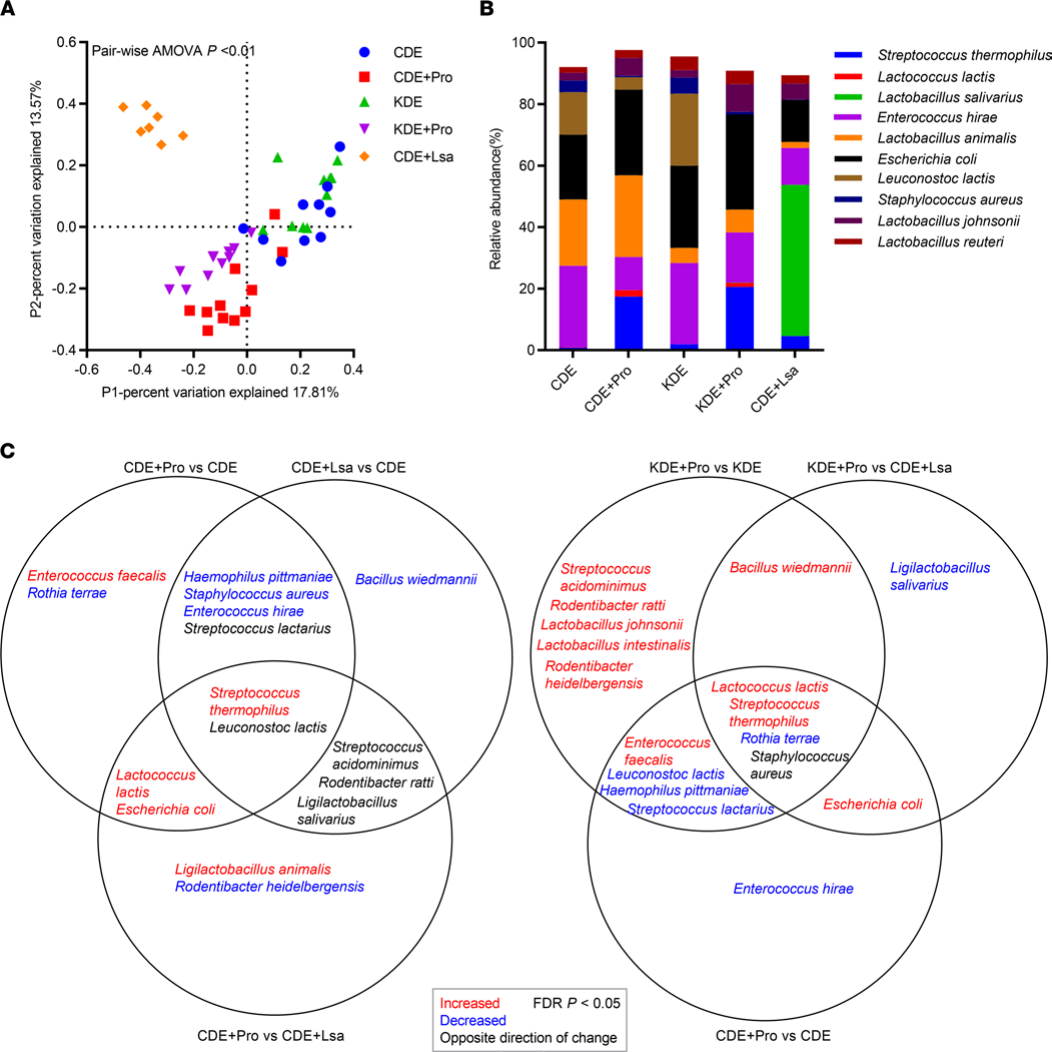
Effects of targeted and negative control probiotic on fecal microbiota composition. (**A**) Principal coordinate analysis. (**B**) Relative abundances of dominant OTUs. (**C**) Venn diagram shows the significantly changed OTUs from pair-wise comparisons. *n* = 10, 11, 10, 11, and 7 for CDE, CDE + Pro, KDE, KDE + Pro, and CDE + Lsa, respectively.

Fecal microbiota profiling validated the impact of oral probiotic colonization. The microbiota composition was distinct across groups (pair-wise AMOVA, *P* < 0.01, Figure 6A). At the species level, the between-group differences were reflected by the relative abundances of dominant taxa (Figure 6B), driven by *S*. *thermophilus* and *L*. *lactis*, *L*. *animalis*, and *L*. *salivarius*. Compared with CDE, CDE + Pro and CDE + Lsa showed some similar effects with decreases in *S*. *aureus* and *E*. *hirae* and with increases in *S*. *thermophiles* (*P* < 0.05). However, *L*. *lactis* was solely increased in CDE + Pro (Figure 6C). Analysis of the predicted metagenomics function showed that both CDE + Pro and KDE + Pro upregulated arachidonic acid metabolism compared with CDE (*P* < 0.05) and numerically increased those involved in ascorbate and aldarate metabolism, folate biosynthesis, and vitamin B6 metabolism (Supplemental Figure 3).

**Figure 7:**
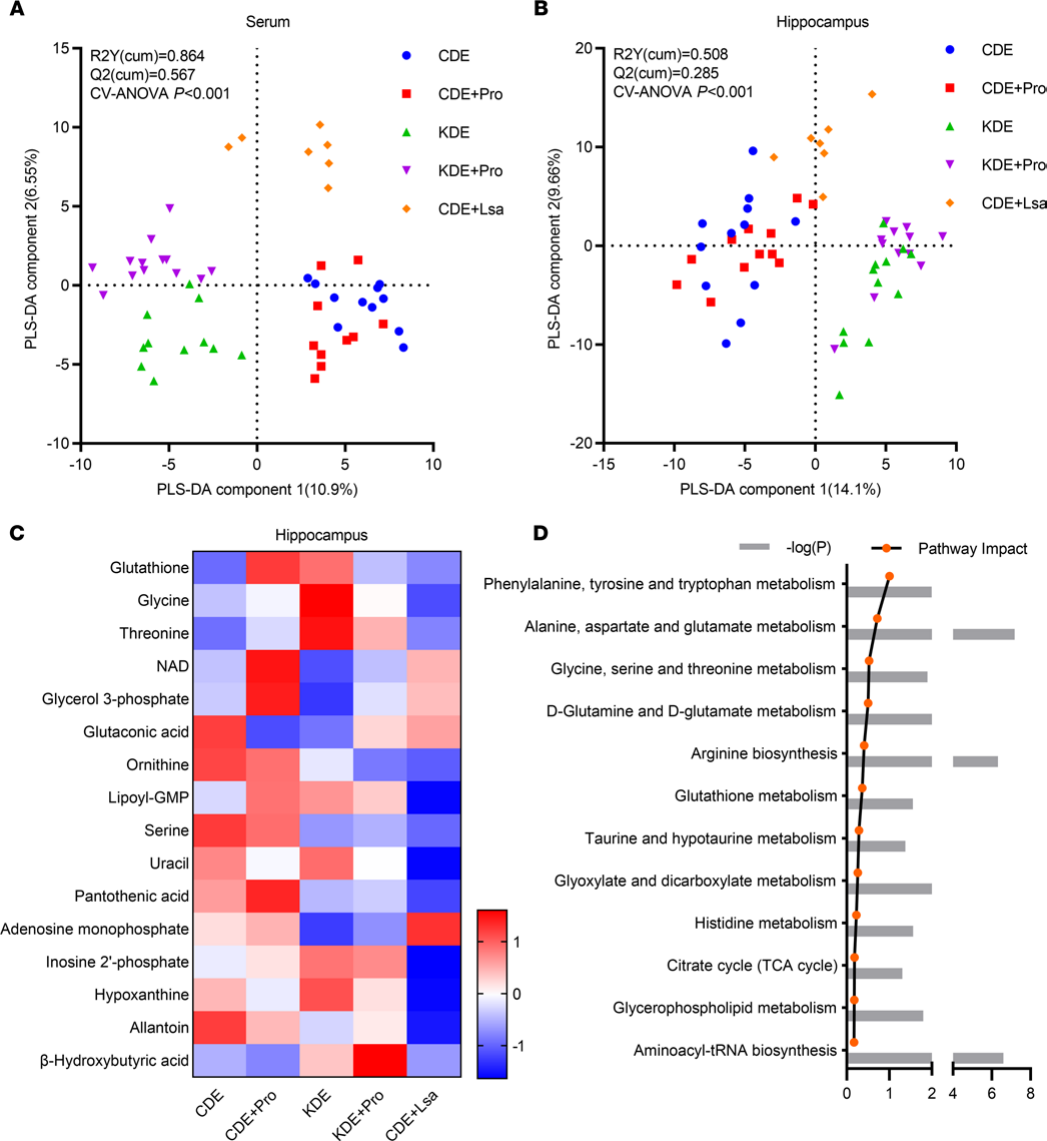
Effects of targeted and negative control probiotic on metabolism. (**A** and **B**) PLS-DA analysis of metabolite composition in serum (**A**) and hippocampus (**B**). (**C**) Heatmap of discriminant metabolites in hippocampus. (**D**) Pathway analysis of hippocampal discriminant metabolites. For serum, *n* = 11, 10, 11, 13, and 7 for CDE, CDE + Pro, KDE, KDE + Pro, and CDE + Lsa, respectively. For hippocampus, *n* = 12, 12, 12, 12,and 7 for CDE, CDE + Pro, KDE, KDE + Pro, and CDE + Lsa, respectively.

Next, we analyzed the metabolomic profiles in both the serum and hippocampus. The hippocampus was targeted because of the well-established association between epilepsy susceptibility and this brain structure (17). In the serum, CD and KD administration led to distinct clusters, with CDE + Lsa clustered separately (CV-ANOVA, *P* < 0.001, Figure 7A). In the hippocampus, treatments also showed significant impact on the overall metabolic profiles (CV-ANOVA, *P* < 0.001, Figure 7B). Here, probiotic treatment altered the relative concentrations of metabolites involved in amino acid metabolism (serine, threonine), glutathione metabolism (glycine, ornithine, glutathione), and purine metabolism (allantoin, hypoxanthine, inosine 2’-phosphate, adenosine monophosphate) (*P* < 0.05, Figure 7C). Pathway analysis showed the enrichment of metabolites involved in phenylalanine, tyrosine, and tryptophan metabolism, as well as glycine, serine, threonine, and glutathione metabolism (*P* < 0.05, Figure 7D).

### Effect of the probiotics on systemic immune signatures.

**Figure 8:**
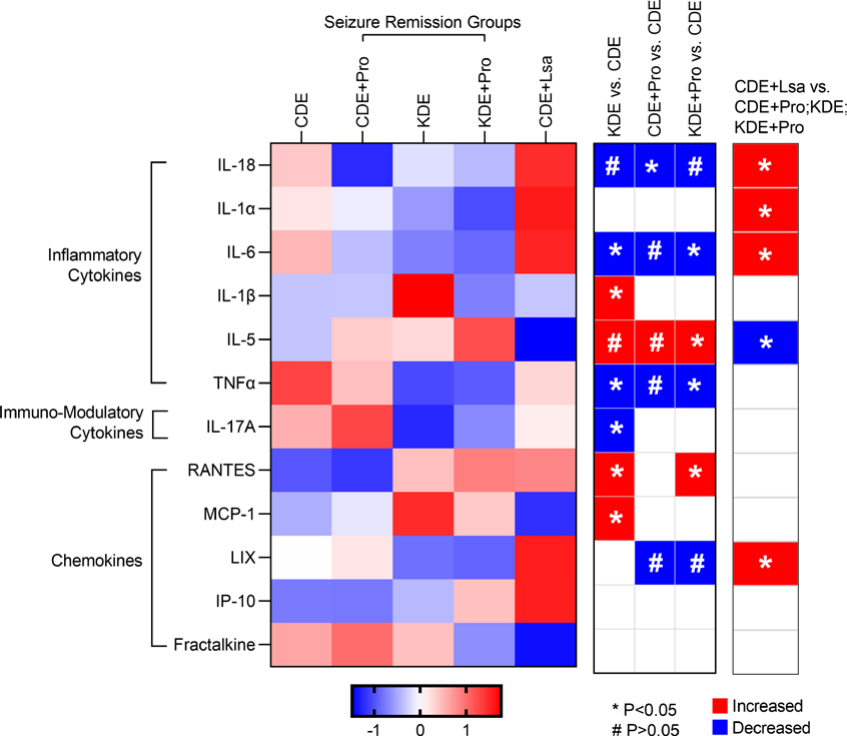
Levels of cytokines and chemokines following targeted and negative control probiotic administration. *n* = 10, 10, 10, 10, and 7 for CDE, CDE + Pro, KDE, KDE + Pro, and CDE + Lsa, respectively. *P < 0.05, ^#^0.05 < *P* < 0.1.

Since inflammation is an integral part of IS (18), and since probiotics may influence immune parameters, we measured the concentrations of major cytokines and chemokines in the serum. As shown in Figure 8, a series of cytokines and chemokines were affected. In parallel with the decreasing number of seizures, CDE + Pro, KDE, and KDE + Pro (seizure remission groups) had lower concentrations of IL-18 (*P* = 0.014, 0.354, and 0.205, respectively), IL-6 (*P* = 0.121, 0.05, and 0.02, respectively), and higher IL-5 (*P* = 0.347, 0.416, and 0.035, respectively), all of which showed opposite direction in the negative control CDE + Lsa (no seizure remission) compared with CDE. Both KDE and KDE + Pro downregulated the proinflammatory marker TNF-α compared with CDE (*P* = 0.018 and 0.023, respectively). CDE + Pro had lower concentrations of IL-1α (*P* = 0.043) and IP-10 (*P* = 0.013) than CDE + Lsa.

## Discussion

The gut microbiota is emerging as a key player in epilepsy, influencing the onset and frequency of seizures (8, 19). While there is growing evidence concerning the role of the diet and the gut microbiota in adult epilepsy, less is known about specific pediatric epilepsy subtypes, including IS. IS is distinct, as it occurs early in life when the microbiota is relatively immature. Our results indicate that the KD changes the gut microbiota and host metabolism, and that some of these alterations can be effectively recapitulated by administration of a targeted probiotic.

The KD is employed clinically around the globe to treat drug-resistant epilepsy in both children and adults. While the mechanism of action of the KD has been a subject of intense scientific interest, emerging preclinical work now shows it to be highly dependent on the gut microbiota (8, 20, 21). Herein, we extend current knowledge to include IS, demonstrating the KD to effectively mitigate seizures compared with a control diet. Analysis of the gut microbiota revealed an increase in the ACE index, an estimate of species richness and α-diversity. This finding is discordant with other studies showing either no change (22) or a decrease in α-diversity with the diet (23). At the phylum level, Actinobacteria was reduced with KD administration, replicating previous work showing it to progressively decline with decreasing carbohydrate in the diet (24). Specific to IS animals, the KD increased relative abundances of *S*. *thermophilus* while decreasing those of *L*. *animalis* compared with the control diet.

Microbial changes associated with the KD were accompanied by an induction of serum metabolites involved in lipid processing (taurocholic acid) and ketogenesis (β-hydroxybutyric acid, acetoacetic acid). Additionally, the KD upregulated serum and hippocampal metabolites involved in antioxidant and antiinflammation processes, such as glutathione, glycine, and *N*-acetyldopamine. Earlier studies have found that patients with epilepsy have a reduced level of cerebral glutathione (25) and that the KD increases glutathione in the brain of patients with intractable epilepsy (26). We also note an increase in *N*-acetyldopamine in both the serum and hippocampus after the KD. *N*-acetyldopamine is a dopamine derivative with antioxidant and antiinflammation activities that reduce superoxide and TNF-α in cells exposed to a stimulus (27). It is possible that the increase of antioxidant status contributes to seizure mitigation following the KD. Taken together, these findings indicate that KD-induced antioxidant upregulation is likely consistent across different epilepsy subtypes, including IS.

To gain further insights into the role of the gut microbiota in IS, antibiotics were administered. Antibiotics further reduced seizure frequency in our model, which was a somewhat unexpected finding. Examination of the fecal microbiota showed increases in *S*. *thermophilus* and *L*. *lactis* with antibiotic administration, with a corresponding reduction in spasms. These microbes are also known to exert psychotropic effects by regulating brain activity and reducing inflammation (28, 29). Likewise, maternal exposure of *L*. *lactis* reduced anxiety-like behavior and improved the development of cortical architecture in mice, which may be involved in immunological regulation by the microbe (30). However, we cannot rule out the microbiota-independent and other off target effects of antibiotics on seizure protection. Antibiotics such as metronidazole can penetrate the blood-brain barrier, especially in young infants (31), while β-lactam–based compounds may upregulate brain synaptic glutamate transporters that, in turn, alter neuronal signaling and seizure potential (32). These and other off-target impacts should be explored in future studies.

It should be noted that the microbes identified in this study are distinct compared with the work of Olson et al*.* (8), who identified *Akkermansia muciniphila* and *Parabacteroides* spp. in adult mice with refractory epilepsy. However, there are several important differences between the aforementioned work and the present study that need to be considered. First, gut microbiota species implicated in adults are rarely present in neonates. Members of *Akkermansia* and *Parabacteroides* species are rarely detected in the infant gut microbiota, which is premature and dominated by *Firmicutes* and *Proteobacteria*, in both humans and rodents (33, 34). Since IS typically manifests early in life when the gut microbiota is still developing, it is not surprising that different microbes might play a role in IS and adult epilepsy. Second, there are likely differences in the permeability of the blood-brain-barrier to bacteria-derived metabolites between infants and adults. Data in the present study were generated in young, P4–P8 rats whose efferent projections are not developed and who have a leaky blood-brain barrier. The blood-brain-barrier is not firmly established in these animals until P16 (35), corresponding to approximately 1 year of age in children. Given these considerations, findings highlight the need to study different epilepsy subtypes, including IS as well as the developmental stage of animals when examining the gut microbiota.

A potentially novel finding of the present study is the creation of a targeted probiotic that recapitulates some of the metabolic and immunological benefits conferred by the KD. Employing data obtained from both KD and antibiotic experiments, we determined that the combination of *S*. *thermophilus* and *L*. *lactis* were strong candidates. These microbes were increased in our manipulations, strongly correlated to one another, and have documented psychobiotic properties (15). Notably, we show that administration of this mixture in a 5:1 ratio, a level observed in the feces, ameliorated seizures and improved locomotor behavior in our model. This finding is strengthened by our use of a negative control, *L*. *salivarius*, that had no effect on seizures or behaviors and supports the species-specific regulation by our selected gut microbes. At present, the independent (single species) as well as the synergistic interactions of these 2 probiotic species on IS tested are not known. In summary, the observed improvements by a targeted probiotic on infantile spasms pave the way for a potentially new therapy involving microbiota manipulation.

In control diet animals with IS, probiotic administration induced some of the metabolic and immunological benefits conferred by the KD, such as increases in serum glutathione and decreases in proinflammatory cytokines including IL-6, IL-18, and TNF-α. Differences were also observed in the hippocampus, with probiotics inducing antioxidant and antiinflammatory pathways. From the present data, we are unable to discern whether changes in cytokines were a cause or were a result of reduced spasms. However, based on the ability of selected probiotics to suppress chronic inflammation and oxidative damage (21, 36), we suspect that the administered probiotics may ameliorate inflammatory responses, contributing to the observed anticonvulsant effects.

Comparable with the KD, targeted probiotics increased glutathione and glycine in our control diet animals. Upregulation of antioxidant processes are thought to be favorable for bioenergetic reserve capacity and reducing reactive oxygen species, both of which may help to stabilize synaptic functions (37). This may be due to an upregulation of microbes involved in ascorbate and vitamin B6 metabolism, each of which can regulate glutathione metabolism and antioxidant status (38, 39). These lines of evidence strongly support a relationship between glutathione metabolism and the gut microbiota in IS. Additional metabolites of interest include increases in nicotinamide adenine dinucleotide (NAD) and glycerol 3-phosphate solely in control diet animals administered probiotics compared with those without. Glycerol 3-phosphate could be used to rapidly regenerate NAD^+^ in the brain (40). NAD^+^ is essential for maintaining ion channel activities, maintaining mitochondrial health, and reducing oxidative stress (41). NAD^+^ treatment reduces spontaneous recurrent seizures by reducing neuronal loss in the CA1 region of the hippocampus in a mouse model (42). Additionally, the decrease of proinflammatory cytokines implicates an immunological mechanism by which probiotics may affect seizure occurrence. Overall, alterations of these metabolic and inflammatory signatures suggest potential mechanisms through which probiotics may be protective against epileptic seizures.

Limitations of this study include the use of 16S rRNA gene sequencing, metagenomics sequencing would have provided a more comprehensive picture of microbial structure and function. In addition, our study design did not examine temporal seizure patterns but, rather, a limited time frame (4 hours/animal). Given this, it is possible that the temporal effects of seizures were missed — a limitation that should be addressed in future studies. Next, the ability of the probiotic to reduce spasms should also be replicated in another drug-responsive model of IS. However, few other models besides the multiple-hit IS model employed here are drug responsive (43). Along these lines, future work could also perform fecal transplant experiments to mechanistically implicate the microbiota dependency of the targeted probiotic.

In conclusion, our studies indicate that specific members of the neonatal gut microbiota have the capacity to regulate seizures, regulate behavior, and shift the metabolic landscape toward an antioxidant status in a model of IS. Together, these findings strengthen the potential therapeutic role of microbiota manipulation by probiotic administration as a potentially novel and complementary approach to prevent seizures and improve developmental outcomes in IS.

## Methods

### Animals.

All animal experiments were conducted on offspring of Sprague-Dawley rats (Charles River Laboratories). The neonatal model of symptomatic IS has been well established as described before (11, 12). Briefly, the day of birth was considered as P0, and on P3, litters were culled to 8 animals. Animals were divided into 2 experimental groups: sham animals that received saline, or epilepsy induction who received doxorubicin/LPS/p-chlorophenylalanine. On P4, intracerebral infusions of doxorubicin and LPS were done stereotaxically under hypothermic anesthesia using the stereotaxic frame (Leica Biosystems). Doxorubicin was injected into the right lateral ventricle followed by LPS into the right parietal cortex (12). The following coordinates were used. Doxorubicin (5 μg/2.5μL): 2.68 mm anterior to lambda, 1.1 mm lateral to sagittal suture, 3.3 mm deep; LPS (3 μg/1.5μL): 2.55 mm anterior to lambda, 1 mm lateral to sagittal suture, 1.7 mm deep. In the morning at P5, animals were injected with p-chlorophenylalanine 200 mg/kg i.p. Sterile saline was used as the vehicle for the 3 injections. Animals were individually placed in beakers warmed in a water bath (45°C) and filled with bedding material (31°C–33°C). Artificial rearing was started once animals had fully recovered from the anesthesia. Animals were continuously fed either a control milk diet (CD, 1.7:1 of fats/carbohydrate + protein) or an isocaloric KD (4:1 of fats/carbohydrate + protein) from P4 to P8. This limited experimental time frame was chosen due to ethical reasons including the highly invasive nature of the surgery and its severe developmental outcomes in animals. It is also a period when animals experience a high rate of seizures and when the antiseizure impacts of the KD mimics what is observed in children with seizure reductions in the range of 50% (3, 11). The milk infusion rate was calculated daily based on the average body weight of the groups. The milk volume was increased by 2% each day. Detailed nutrient ingredients can be found in Supplemental Table 1.

### Antibiotic treatment.

On P5, animals described above were randomly assigned to the control or antibiotic treatment. An Abx mixture was added to formula at the daily dosage of 20 mg/kg vancomycin (V2002, MilliporeSigma), 50 mg/kg neomycin (N6386, MilliporeSigma), 50 mg/kg ampicillin (A9393, MilliporeSigma), and 10 mg/kg metronidazole (M3761, MilliporeSigma). These dosages were chosen based on therapeutic levels given to infants and were designed to deplete gut microbiota (44, 45). The dosage given was converted based on the milk speed during artificial rearing and the body weight per day to ensure the corresponding amount of antibiotics being reliably administered. The resulting experimental groups in the antibiotic experiment were CDE, CDE + Abx, KDE, and KDE + Abx.

### Targeted probiotic and negative control administration.

*S. thermophilus* HA-110, *Lactococcus* *lactis* subsp. *lactis* HA-136, and *Ligilactobacillus salivarius* HA-118 (previously described as *Lactobacillus salivarius*) (46) were provided as pure strains by Lallemand Health Solutions. Microbes were freshly rehydrated in double-distilled water at 1 × 10^10^ CFU per mL. A total of 100 μL or water or probiotic was gavaged per animal, per day. *S*. *thermophilus* HA-110 and *L*. *lactis* HA-136 were mixed in a 5:1 ratio based on findings discussed below. The resulting experimental groups were as follows: CDE, CDE + Pro, KDE, and KDE + Pro. A negative control was also included (CDE + Lsa).

### Seizure quantification.

Seizures characterized by rapid extension and flexion movements were recorded in a blind manner using a video system with Sirenia software (Pinnacle Technology) following published methods (12). A total of 4 hours of recordings on P7 were quantified per animal. Spasms were quantified by date and by a single reviewer, who was blinded to the treatments.

### Neurobehavioral testing.

Surface righting, negative geotaxis (47), and open-field tests (11) were performed in a blind manner as previously described on P7. For open-field test, the animal was placed in the center of a box (20 cm length × 20 cm width × 25cm height) above a camera equipped with a tracking system (TopScan tracking) and was allowed to move freely for 5 minutes. Animal movements were quantified with Ethovision XT software.

### Blood and serum measurements.

Blood concentrations of glucose and ketones were measured with FreeStyle Precision Neo meters (Abbott Laboratories). Cytokine and chemokine measurements were performed on serum, 2-fold diluted with PBS (pH 7.5) and assessed by a Rat Cytokine and Chemokine Array 27 Plex by Eve Technologies.

### Bacterial genomic DNA extraction and 16S rRNA high-throughput sequencing.

Fecal genomic DNA was extracted using a FastDNA SPIN Kit for Feces (MP Biomedicals) and quantified using a high-sensitivity dsDNA Qubit Kit (Invitrogen). The DNA library construction and sequencing were performed on an Illumina MiSeq platform with the MiSeq V3 600 cycle sequencing kit in the Core DNA facility at the University of Calgary. The sequence demultiplexing and removal of indices were performed using the bacterial metagenomics workflow in the MiSeq Reporter software (Illumina). Sequences were then processed with Mothur Version 1.35.1 following the online MiSeq standard operating procedure (48). Sequence reads were further analyzed using the 16S Metagenomics app (version 1.0.1; Illumina) for taxonomy assignment. A total of 180,322 ± 8580 (mean ± SEM) reads were obtained per sample. After quality control and filtering, 74,250 ± 3597 (mean ± SEM) reads were obtained per sample. To compare samples on an equal basis, all samples were rarefied to even sampling depths at the lowest observed depth. To obtain species-level resolution, representative sequences of each OTU were also loaded into the National Center for Biotechnology Information Basic Local Alignment Search Tool website against the 16S rRNA-Seq database. The metagenomics prediction of 16rRNA data were conducted using PICRUSt (49). 16S rRNA-Seq data are available via the NCBI Sequence Read Archive (SRA PRJNA796474, PRJNA795716).

### Quantitative PCR.

Quantification of total bacteria was performed in a 7500 Fast Real-Time PCR Systems (Applied Biosystems) following previously described methods (50).

### Metabolic fingerprinting with LC-QTOF-MS.

A total of 50 μL of serum and approximately 10mg of hippocampal tissues (wet weight) were used for sample preparation following previously reported methods (51, 52). Samples were analyzed using an Agilent 6550 iFunnel Q-TOF LC/MS system (Agilent) with an Acquity UPLC HSS T3 column (53). Raw data were converted to mzXML format by ProteoWizard 3.0 package and uploaded to XCMS online (54). Metabolites were identified against METLIN (55) and Human Metabolome Database (56). Multivariate analysis was performed with SIMCA 13.0 (Umetrics). VIP scores were assessed. Discriminated metabolites were defined with a VIP > 1.0 by partial least squares-discriminant analysis (PLS-DA), *P* < 0.01, FDR-adjusted *P* < 0.05. Pathway enrichment analysis were analyzed using Metaboanalyst 4.0 (57).

### Statistics.

Data are presented as mean ± SEM. Comparisons between 2 groups were conducted using a nonparametric Mann-Whitney *U* test or 2-tailed Student’s *t* test, depending on the normality of data distribution. For the time-series data of body weight gain, 2-way ANOVA with repeated-measure analysis was applied to compare the effects of age or treatment on body weight gain. For data of seizure frequency, α-diversity, bacterial 16S rRNA gene copies, behavior readouts, and ketone and cytokine concentration, 1-way ANOVA with Tukey’s post hoc was used to determine differences between groups. Two-way ANOVA (diet × treatment) and 3-way ANOVA (epilepsy × diet × antibiotic treatment) were also employed to test the effects of variables. Comparisons of taxa were analyzed by Kruskal-Wallis 1-way ANOVA with Dunn’s post hoc test (GraphPad Software). Taxonomic data was analyzed using LEfSe (58) to identify microbial signatures. An FDR correction was applied for multiple tests where applicable. Correlation analysis was conducted using a Spearman’s correlation. *P* < 0.05 was considered significant. Sample size was determined to achieve a power of 0.8 for ANOVA test using the G*Power 3.1.9.2.

### Study approval.

All procedures were performed under guidelines of the Canadian Council on Animal Care and had ethics approval by the Health Sciences Animal Care Committee at the University of Calgary (AC20-0013).

## Author contributions

CM and JS designed and conducted experiments, performed data acquisition, analysis and interpretation, and wrote the manuscript. NN and TAT contributed to provide new reagents. AC, MW, and WNM participated the research. NN, TAT, JMR, and MHS participated in the manuscript writing. JS conceived and supervised the study.

## Supplementary Material

Supplemental data

## Version 1 06/22/2022

Electronic publication
