# Supplementary material for: Targeted gut microbiota manipulation attenuates seizures in a model of infantile spasms syndrome
Source: JCI Insight. 2022 Jun 22;7(12):e158521. doi: 10.1172/jci.insight.158521 (PMC9309045; doi:10.1172/jci.insight.158521)
Supplement: Supplemental data [file jciinsight-7-158521-s234.pdf]

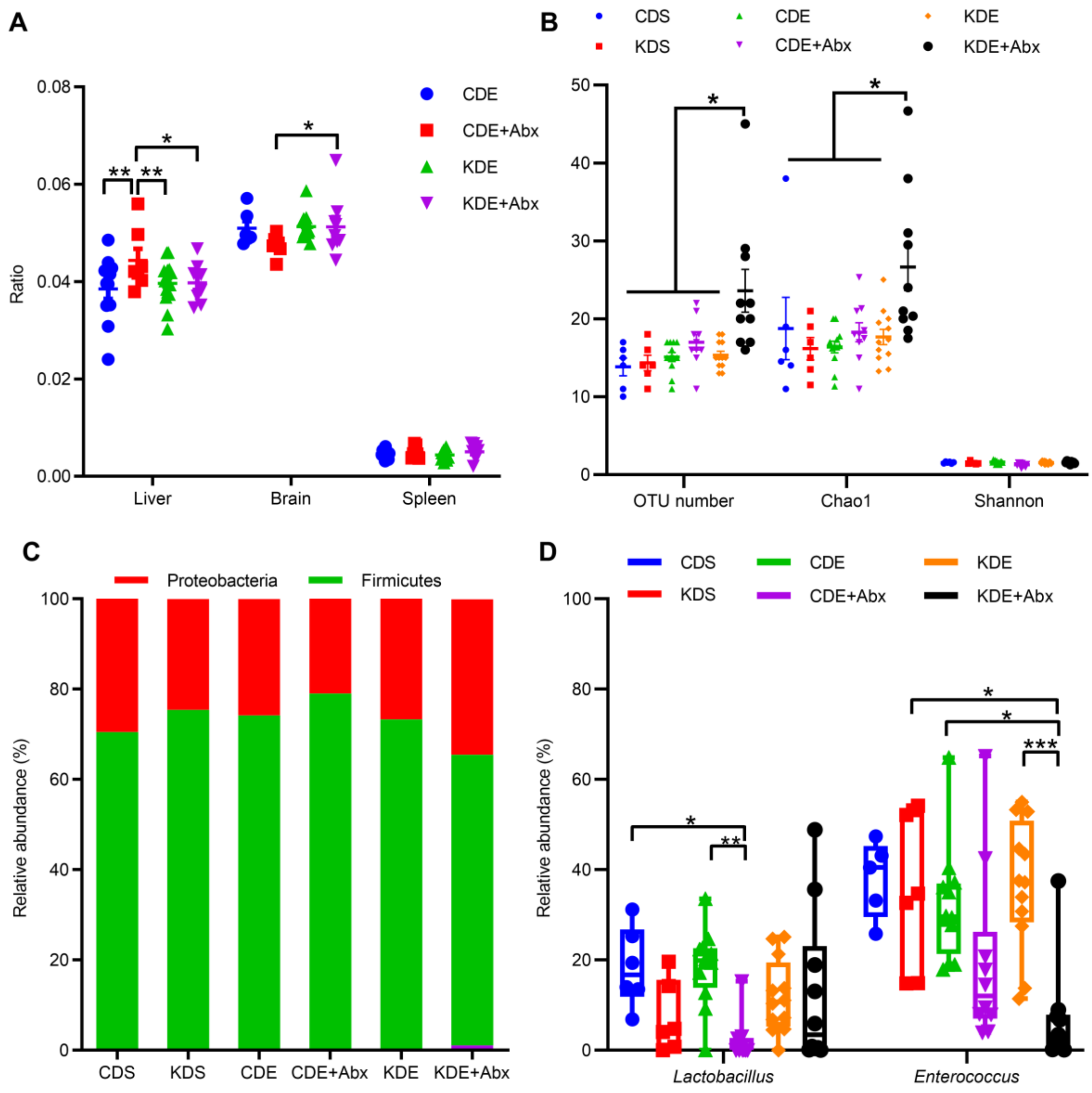

Supplemental Figure 1 Effects of antibiotics on microbial diversities and specific taxa. (A) Relative organ mass (n=6-16/group). (B) α-diversity (n=6-12/group). (C) Microbial composition at the phylum level (n=6-12/group). (D) The relative abundances of *Lactobacillus* and *Enterococcus* (n=6-12/group). The data was analyzed using One-way ANOVA with Tukey's post hoc (A) or Kruskal–Wallis ANOVA with Dunn's post-hoc test (B, D). Figures S1A, S1B, S1D, values are mean ± SEM. Figure S1C, values are means. Superscripts indicate significant difference between groups. *p<0.05, **p<0.01, ***p<0.001.

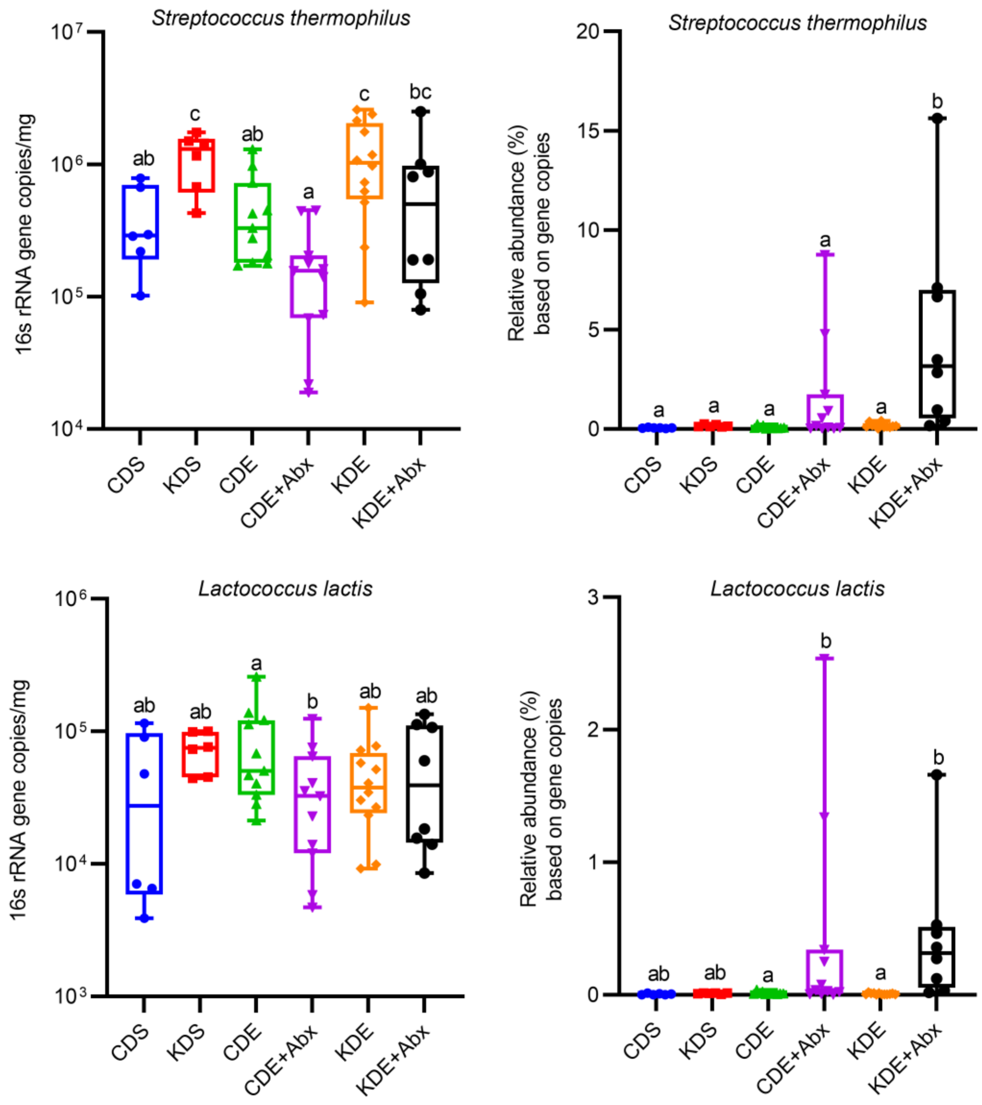

Supplemental Figure 2 Quantitative PCR analysis of fecal 16S rRNA gene copies of Streptococcus thermophiles and Lactococcus lactis (n= 6, 6, 11, 11, 12, 8 for CDS, KDS, CDE, CDE+Abx, KDE, and KDE+Abx, respectively). The relative abundance was calculated by using the 16S rRNA gene copies per taxa divided by the total 16S rRNA gene copies. The data was analyzed using One-way ANOVA with Tukey's post hoc. Values are mean ± SEM. Labelled means without a common superscript letter differ, $p<0.05$.

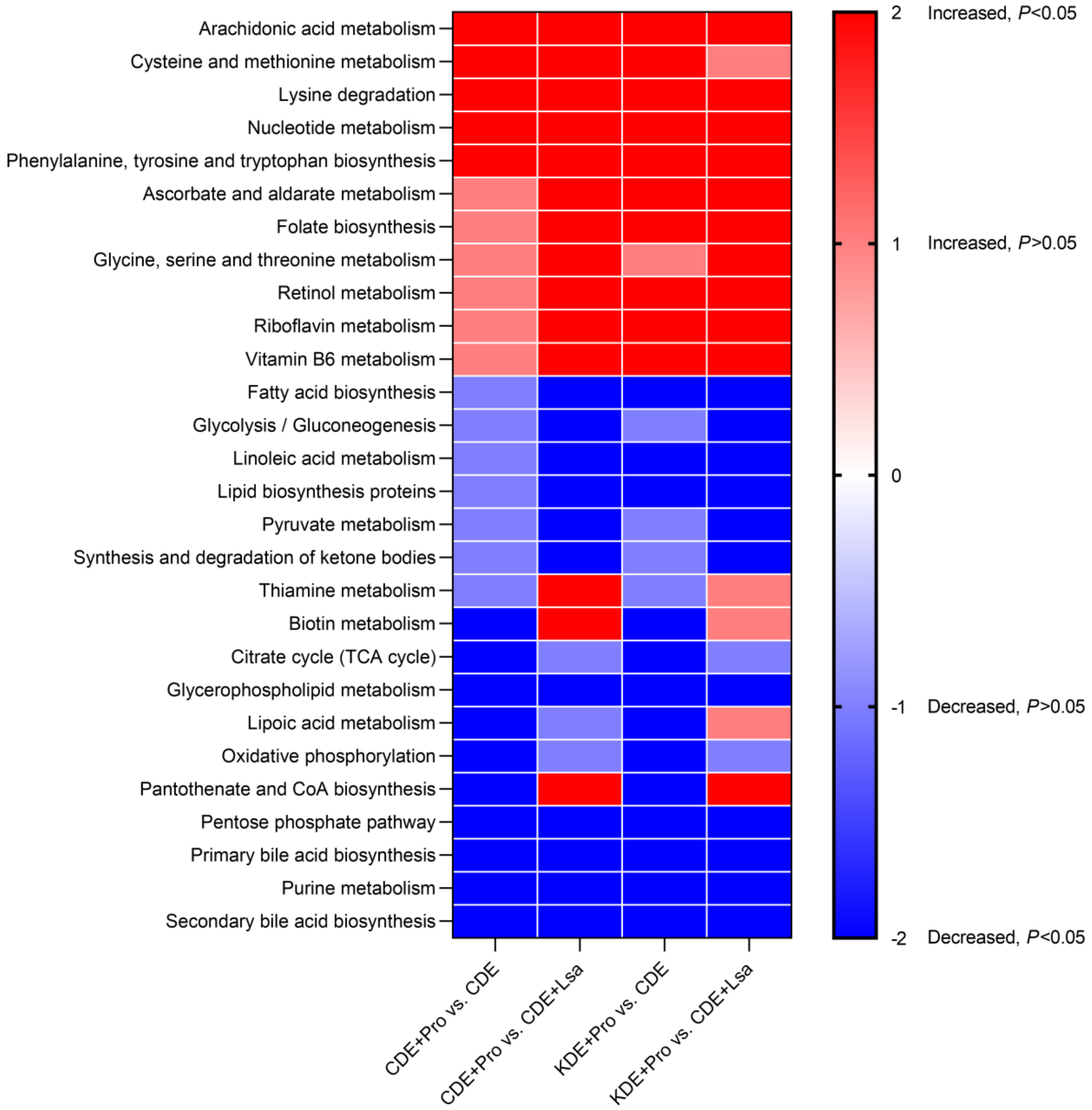

Supplemental Figure 3 Metagenomics functional prediction of probiotic-induced microbiome alterations. Values are represented as fold changes in the heatmap.

Ingredients	Control Rat Milk Formula Amount (g/Kg)	Ketogenic Rat Formula Amount (g/Kg)
**Calories Total**	2713 kcal	2657 kcal
Fat Total	233.25	264.75
Saturated	34.92	31.23
+Trans	0	0
Polyunsaturated	99.65	129.84
Omega-6	129.55	168.79
Omega-3	1.99	2.60
Monounsaturated	49.83	64.92
Cholesterol	0.19	0.11
Carbohydrate	105.66	20.41
Fibre	0	0
Sugars	27.15	5.79
Protein	59.39	55.83
**Amino Acid Profile (EAA: Essential Amino Acids, BCAA: Branded-Chain Amino Acids)**		
Alanine	4.11	6.91
Arginine	2.70	4.54
Aspartic Acid	9.87	16.58
Cysteine	2.0	3.36
Glutamic Acid	15.63	26.25
Glycine	1.65	2.76
Histidine	1.88	3.16
Isoleucine BCAA	5.41	9.08
Leucine BCAA	10.34	17.37
Lysine EAA	1.88	3.16
Phenylalanine EAA	3.06	5.13
Proline	7.76	13.03
Serine	5.41	9.08
Threonine EAA	7.76	13.03
Tryptophan EAA	2.23	3.75
Tyrosine	3.17	5.33
Valine EAA BCAA	6.11	10.26
**Vitamin and Mineral Composition**		
Sodium	0.67	0.39
Calcium	120.65	43.15
Iron	0.004	0.003
Linoleic acid	3.20	2.85
Aspartame	0.017	0.015
Thiamine	0.00022	0.00019
Riboflavin	0.00022	0.00020
Niacin	0.0026	0.0023
Folic Acid	6.59E-05	5.88E-05
Pantothenic Acid	0.00090	0.00080
Biotin	4.33E-06	3.87E-06
Choline	0.17	0.15

Inositol	0.047	0.042
Phosphorus	0.23	0.20
Magnesium	0.038	0.034
Zinc	0.0020	0.0018
Iodine	2.84E-05	2.54E-05
Manganese	0.00055	0.00050
Copper	0.00028	0.00025
Molybdenum	9.01E-06	8.05E-06
Chromium	6.59E-06	5.88E-06
Selenium	1.18E-05	1.05E-05
Potassium	0.37	0.33
Chloride	0.24	0.15
Vitamin A	519.93 IU	464.32 IU
Vitamin C	108.61 %DV + 0.02g	23.16 %DV + 0.018g
Vitamin B6	0.00022	0.00020
Vitamin B12	4.51E-07	4.024E-07
Vitamin D	162.91 %DV	34.74 %DV
Vitamin D3	72.10 IU	64.39 IU
Vitamin E	199.31 %DV + 3.81 IU	259.67 %DV + 3.40 IU
Vitamin K	1.39E-05	1.24E-05

Supplemental Table 1. Detailed composition of control and ketogenic diets. Diets are based on Nutricia KetoCal Formulation (see: https://shop.myketocal.com/product/ketocal-41-powder) for detailed [illegible]
